# Harnessing Implementation Science in Clinical Psychology: Past, Present, and Future

**DOI:** 10.1146/annurev-clinpsy-081423-021727

**Published:** 2025-02-19

**Authors:** Rinad S. Beidas, Meredith Boyd, Elizabeth Casline, Kelli Scott, Zabin Patel-Syed, Chynna Mills, Brian Mustanski, Simone Schriger, Faith Summersett Williams, Claire Waller, Sarah A. Helseth, Sara J. Becker

**Affiliations:** 1 Department of Medical Social Sciences, Feinberg School of Medicine, Northwestern University, Chicago, Illinois, USA; 2 Center for Dissemination and Implementation Science, Institute for Public Health and Medicine, Feinberg School of Medicine, Northwestern University, Chicago, Illinois, USA; 3 Department of Psychiatry and Behavioral Sciences, Feinberg School of Medicine, Northwestern University, Chicago, Illinois, USA; 4 Department of Psychology, University of Pennsylvania, Philadelphia, Pennsylvania, USA; 5 Department of Pediatrics, Feinberg School of Medicine, Northwestern University, Chicago, Illinois, USA

**Keywords:** implementation science, behavior change mechanisms, improving quality of behavioral health care

## Abstract

Implementation science aspires to equitably accelerate the uptake of clinical research into practice to improve population health. The focus of implementation science includes individual behavior change mechanisms that are similar to those that drive the field of clinical psychology. For this reason, clinical psychologists are well-suited to take up implementation science methods in pursuit of improving the quality of behavioral health care. To do so, clinical psychologists must expand beyond individual behavior change to include a focus on organizations and systems. In this review, we reflect on ways that clinical psychologists can lead in the integration of implementation science principles and approaches into clinical psychology research and practice. We discuss the role clinical psychologists play in closing know–do gaps in behavioral health and describe how clinical psychologists can build implementation science competencies. We end with current controversies and opportunities for innovation to further improve the quality of behavioral health care.

## INTRODUCTION

The United States is a global leader in spending on behavioral health research but ranks far behind other nations in overall well-being and behavioral health outcomes (Curtin et al. 2023). Nine in ten Americans believe the United States faces a behavioral health crisis (CDC Newsroom 2023), exacerbated by the COVID-19 pandemic, with deaths from suicide and drug overdose at record highs. Billions of dollars are invested in developing and testing behavioral health interventions, while far less is spent on studying the implementation of effective interventions in clinical practice (NIMH 2023). As Tom Insel, MD, former director of the National Institute of Mental Health (NIMH), noted, “The country’s mental health crisis is not a research problem, it is an implementation problem” (cited in Barry 2022).

Currently, it takes decades (Balas & Boren 2000, Khan et al. 2021) for evidence-based treatments (EBTs) for behavioral health to reach clinical practice, a major public health problem known as the know–do gap that perpetuates health inequities and limits the benefits from scientific discoveries in behavioral health. The reasons for this gap are multifaceted and span barriers at the organization, clinician, and client levels (Beidas et al. 2016). To address this gap between what is known and what is done in behavioral health practice, the NIMH, the National Institute for Drug Abuse (NIDA), and other national and international funding agencies have prioritized investing in implementation science (IS).^1^ IS is “the scientific study of methods to promote the systematic uptake of research findings and other evidence-based practices into routine practice, and, hence, to improve the quality and effectiveness of health services” (Eccles & Mittman 2006). IS aims to develop generalizable knowledge about effective methods to equitably increase the availability and sustainment of EBTs, with the end goal of improving population health. IS prioritizes equity and antiracism, recognizing that efforts to expand care access without this focus often reify existing social and health disparities (Brownson et al. 2021, Shelton et al. 2021b). The field has an explicit focus on integrating methods from multiple disciplines, including psychology, organizational and management theory, engineering, anthropology, critical race theory, and public policy, with the goal to equitably close know–do gaps (McHugh & Barlow 2010, Nilsen 2015, Stirman et al. 2016). Broadly, IS focuses on supporting individual behavior change for clinicians, leaders, and clients within organizational or societal constraints (Hodson et al. 2024).

To apply IS methods, clinical psychologists must expand their focus beyond the level of individual treatment delivery to organization and system levels. This multilevel perspective affects all aspects of implementation, including alliance building. Clinical psychologists prioritize collaboration and building therapeutic alliances with clients, while implementation researchers partner with health systems, community organizations, and clinicians. Just as the client–therapist alliance is crucial for therapy success, partnerships between implementation scientists, clinicians, and affected parties are vital for IS initiatives. Successful partnerships hinge on trust, mutual respect, and shared goals, whereas challenges to partnership include funding constraints, struggles for control, and lack of role clarity (Drahota et al. 2016, Pellecchia et al. 2018).

In this article, we review the unique role that clinical psychologists play in leading efforts to close know–do gaps in behavioral health. We first outline steps that clinical psychologists can take to integrate IS methods into their work. We compare these steps to those a clinical psychologist would take to engage, diagnose, treat, and evaluate client progress in therapy in order to draw parallels between the two fields given their shared goals. We then discuss how clinical psychologists new to IS can build competencies. We conclude by considering controversies and opportunities to better integrate IS and clinical psychology to actualize the potential of transforming the quality of behavioral health.

## IMPLEMENTATION FRAMEWORKS, THEORIES, AND MODELS

IS strives to understand why implementation efforts succeed or fail and uses this generalizable knowledge to enhance and predict success. IS frameworks, theories, and models (FTMs) are essential tools in this endeavor. Nilsen (2015) identifies three functions to guide FTM selection relevant to behavioral health efforts. First, FTMs guide the process of implementation. These FTMs outline key steps for implementing and sustaining EBTs (Taylor et al. 2014). A seminal process model, initially developed for public sector organizations, is the Exploration, Preparation, Implementation, Sustainment (EPIS) Framework (Aarons et al. 2011). Table 1 presents definitions of each EPIS phase, steps commonly undertaken during each phase, and conceptual linkages to the therapy process to align with the typical work of clinical psychologists. The four phases of EPIS emphasize understanding the context prior to implementation, the factors impacting implementation over time, and tailored strategies for each phase (Aarons et al. 2011). Second, FTMs aim to explain successful implementation, and these include determinant frameworks, classic theories, and implementation theories. Determinant frameworks are the most widely used to identify contextual factors influencing implementation. Third, FTMs support the evaluation of implementation strategies, assessing the success or failure of initiatives. Seminal determinant and evaluation frameworks are described in subsequent sections of this article.

**Figure 1:**
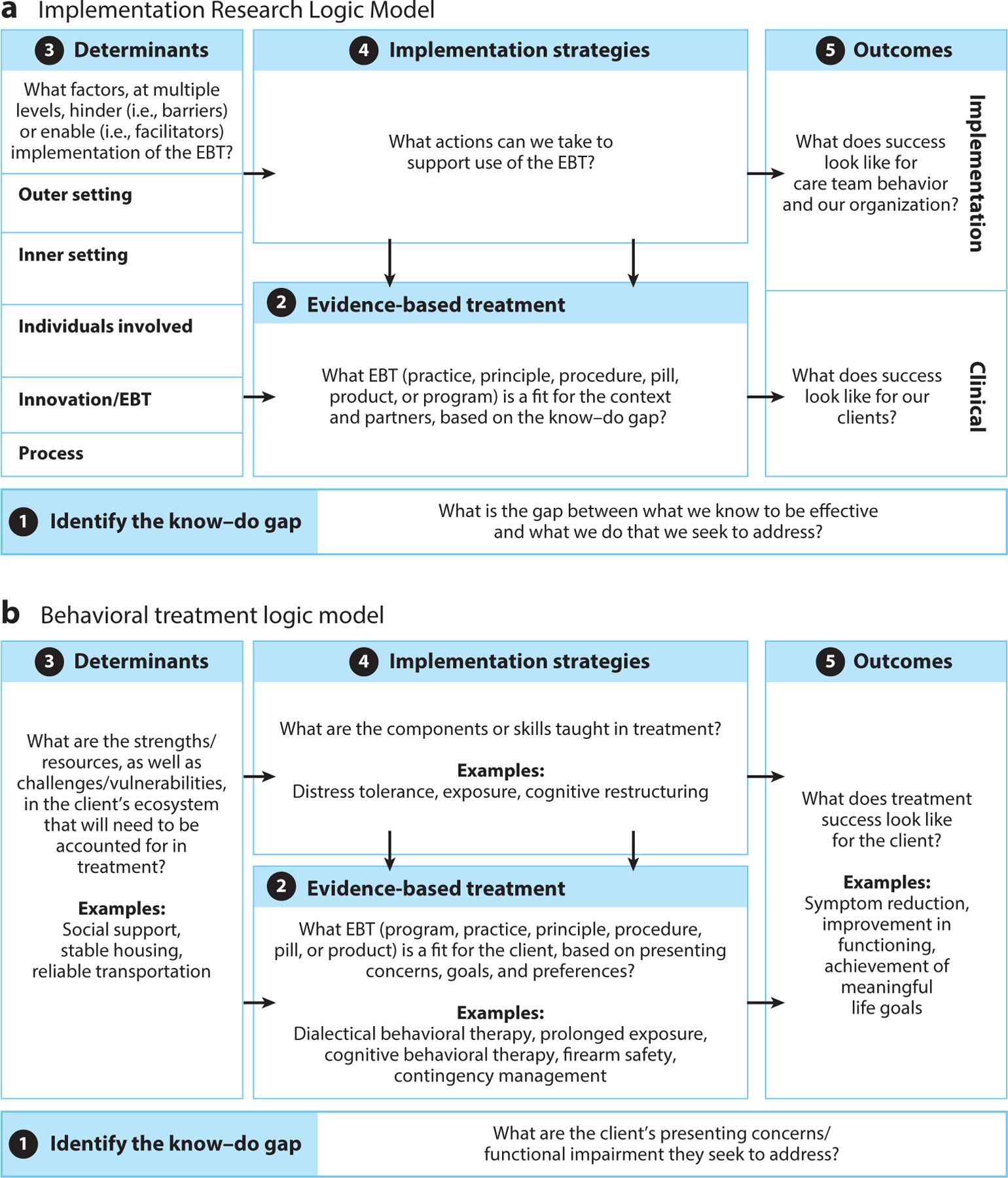
Implementation Research Logic Model and behavioral treatment logic model. Circled numbers indicate steps for moving through the figure. Abbreviation: EBT, evidence-based treatment. Figure adapted from Smith et al. (2020) (CC BY 4.0).

An implementation effort often requires using FTMs that span all three of these functions. The Implementation Research Logic Model (IRLM) (Figure 1a) is a blueprint for integrating multiple FTMs that specifies the relationships between core IS components (Smith et al. 2020). As shown in Figure 1a, each component of the IRLM requires consideration of key questions that can be used to plan, execute, and report on an implementation effort (Smith et al. 2020). While the IRLM can be used flexibly, and elements do not need to be addressed chronologically, we recommend a sequence of steps that can be helpful for clinical psychologists new to IS: (*a*) identify the know–do gap, (*b*) select the EBT, (*c*) identify contextual determinants, (*d*) select implementation strategies to address determinants, and (*e*) select outcome measures to evaluate implementation. Figure 1b depicts how each component of our recommended IRLM sequence parallels fundamental steps of the therapeutic process.

In the next sections, we define each step of the IRLM and introduce key FTMs that can be used to support each activity. We elucidate each step throughout via two illustrative behavioral health case examples: (*a*) implementation of a secure firearm storage program as a universal suicide prevention program in pediatric primary care (see Beidas et al. 2021 for the trial protocol), and (*b*) implementation of contingency management (CM) as an adjunct to medication in opioid treatment programs (see Becker et al. 2021 for the trial protocol).

### Steps 1 and 2: Identify the Know–Do Gap and Select the Evidence-Based Treatment

Two closely related steps in the implementation process are to identify the know–do gap and to select an EBT for implementation (see Figure 1a, Steps 1–2). These steps parallel the initial phases in clinical psychology of conceptualizing and diagnosing a client’s primary presenting concerns, then selecting an appropriate EBT for treatment (see Figure 1b, Steps 1–2). Identification of the know–do gap requires selecting a problem with high public health significance for which solutions exist but are not readily available or accessible. In the same manner that patients must be engaged in the identification of presenting problems, community members must be engaged in the identification of know–do gaps to ensure shared understanding of the public health problem and of the specific context for which the implementation initiative is intended (Drahota et al. 2016, Pellecchia et al. 2018).

The next step is to select EBT(s) that address the identified know–do gap and are well-suited for a particular setting. We use EBT as an umbrella term for evidence-based behavioral health programs, practices, principles, procedures, pills, and products that can be implemented across settings with the goal of improving health (Brown et al. 2017, Lane-Fall et al. 2019). The selection of EBTs can be informed by the answers to a few questions about the existing research evidence. Are there any EBTs with sufficient evidence of efficacy (i.e., effect of the intervention on clinical outcomes in a highly controlled setting that prioritizes internal validity) and effectiveness (i.e., effect of the EBT on clinical outcomes in a setting that better approximates real-world conditions and prioritizes external validity) derived using rigorous methods? Have any EBTs been examined in the target setting and with the population who is intended to benefit from the implementation initiative?

As the field continues to evolve, it is important to acknowledge that there may be times when the evidence is not robust for a particular EBT or know–do gap. For example, although there are over 500 EBTs to address children’s mental health needs alone (Okamura et al. 2020), significant gaps remain, as the current foundation of evidence was built on research conducted in highly controlled contexts (i.e., university based clinics) with participants who may not resemble those who present for care in community settings (e.g., lacking diversity in ethnicity, race, and socioeconomic status and presenting lower comorbidity and clinical acuity; Southam-Gerow et al. 2008). It is therefore possible that a community may select an intervention lacking strong evidence in a specific setting or population because it has encouraging data in other contexts; IS can still be useful in these circumstances.

In addition to evaluating the evidence, it is important to select an EBT that is sensitive to the values, preferences, and needs of the client population, the clinicians who will deliver it, and the organizational leaders who will support implementation. To prevent implementation challenges, there is often a need to adapt an EBT to better meet constituent needs and preferences. Adaptation involves purposeful changes to an intervention or its implementation with the goal of improving effectiveness and contextual fit (Wiltsey Stirman et al. 2019). While an in-depth discussion of adaptation is beyond the scope of this review, resources have been developed to adapt EBTs before implementation and to facilitate rigorous tracking of adaptations to reduce the likelihood of drifting from effective EBT core components (Aarons et al. 2012, Chambers & Norton 2016, Wiltsey Stirman et al. 2019, Wingood & DiClemente 2008). Adaptation methods are common as a precursor to implementation in behavioral health implementation efforts. For instance, a recent systematic review identified 32 studies that adapted 19 trauma interventions (Cavanaugh & Wismar 2022). An alternate approach to resource-intensive adaptation is to begin by explicitly testing promising EBTs in the context in which they are delivered to ensure relevance and fit (Beidas et al. 2023, Kwan et al. 2022). The sidebars titled “Step 1: Identify the Know–Do Gap” and “Step 2: Select an Evidence-Based Practice” present our two behavioral health case examples and the methods employed to select and adapt the EBTs under study.

### Step 3: Identify Contextual Determinants

After identifying the problem, selecting, and adapting (if needed) an EBT, the next step is to identify contextual factors likely to influence the success of implementation (Figure 1a). This step parallels conducting a comprehensive biopsychosocial assessment of the patient to understand the social determinants of health and other factors that are likely to influence the success of the EBT (Figure 1b). Gaining an understanding of determinants prior to implementation helps implementation teams consider how context might relate to implementation and informs the selection of implementation strategies.

Implementation determinants are contextual factors at multiple levels that can have a hindering effect (known as a barrier) or an enabling effect (known as a facilitator) on implementation and client outcomes (Nilsen & Bernhardsson 2019). As noted in the section titled “Implementation Frameworks, Theories, and Models,” determinant frameworks have been developed to guide the evaluation of determinants prior to and during the implementation of new practices (Nilsen 2015, Nilsen & Bernhardsson 2019). One widely used determinant framework, the Consolidated Framework for Implementation Research (CFIR; Damschroder et al. 2009, 2022b), synthesizes constructs from multiple FTMs, making it a comprehensive tool for elucidating determinants, and it also provides guidance including interview guides (CFIR Res. Team 2024), measures assessing specific constructs (Fernandez et al. 2018), and matching of determinants to implementation strategies (CFIR Res. Team 2024). Recently updated, the CFIR (Damschroder et al. 2022b) identifies determinants across five ecological domains: characteristics of the EBT, outer setting (i.e., the broader sociopolitical context in which the organization operates), inner setting (e.g., the community behavioral health organization or health system where the EBT will be delivered), individuals involved (e.g., clinicians or clients receiving or using the EBT), and implementation processes. In recent years, the CFIR has been used to plan for the implementation of EBTs for specialty mental health promotion (Van Deinse et al. 2019), child behavioral health (Barwick et al. 2020), and community-based interventions for depression and anxiety globally (Petersen et al. 2021).

Though CFIR’s comprehensiveness is a strength, the framework was not developed to explicitly center health equity (Woodward et al. 2019), which is critical when engaging in implementation initiatives to ensure that new inequities are not created or reified during the implementation process and to ensure equitable implementation and corresponding health benefits for all people (Baumann & Cabassa 2020, Brownson et al. 2021). The Health Equity Implementation Framework (HEIF; Woodward et al. 2019) was explicitly developed to integrate a health equity perspective into the identification of contextual determinants, bringing together a well-known IS contextual determinants framework, the Integrated-Promoting Action on Research Implementation in Health Services framework (Harvey & Kitson 2016), and the Health Care Disparities Framework (Kilbourne et al. 2006). It includes multiple domains for consideration—namely, clients, clinicians, clinical encounters, health care systems, contexts, recipients, and characteristics of the EBT. Novel aspects of the HEIF include its emphasis on the clinical encounter, or the interactive effect between clinician and client factors that may influence intervention delivery, its consideration of upstream societal effects on downstream determinants, and its expansion of recipient factors to include determinants that are specific to health equity (e.g., a clinician’s knowledge, attitudes, or biases about a particular group; Woodward et al. 2019).

Clinical psychologists seeking to understand the determinants affecting uptake of an EBT prior to implementation can begin by identifying a framework relevant to their efforts, such as the CFIR or HEIF, or by using a determinant checklist, such as the integrated checklist of determinants of practice developed by Flottorp et al. (2013). After selecting the framework, the next step requires deciding which domains are most relevant to the EBT and its implementation, based on the existing literature, the specific implementation effort, and consultation with partners. After domain selection, the next step requires choosing methods for evaluating contextual determinants in the setting where the new practice will be implemented. This typically entails multi-informant, mixed-methods approaches, which may include observation of clinical workflows, qualitative interviews, and validated quantitative surveys with key constituents. In the sidebar titled “Step 3: Identify Contextual Determinants,” we describe the process of identifying contextual determinants most likely to influence implementation in our two behavioral health case examples.

### Step 4: Select Implementation Strategies to Address Determinants

The fourth step of the IRLM is to select strategies to address the identified determinants (see Figure 1a). This step parallels the selection of specific intervention techniques to address the challenges and capitalize on the strengths of the client identified during the psychologist’s biopsychosocial assessment (see Figure 1b).

Implementation strategies are the actions taken to facilitate successful adoption, implementation, and sustainment of EBTs across a range of clinical settings (Proctor et al. 2013). Just as psychological EBTs aim to support patient behavior change, implementation strategies aim to support clinician behavior change within organizational constraints or a broader organizational change. Implementation strategies can therefore be conceptualized as interventions on the target system. Multiple taxonomies exist to help characterize and select client-, clinician-, and organization-level implementation strategies to facilitate practice change, including many that have been used in behavioral health implementation studies.

A frequently used taxonomy to guide implementation strategy selection is the Expert Recommendations for Implementing Change (ERIC; Powell et al. 2015), a set of 73 implementation strategies identified through expert consensus via a systematic Delphi process. ERIC strategies have been mapped onto a pragmatic set of nine categories, to support researchers and implementers in the selection and operationalization of strategies (Waltz et al. 2015). These are: engaging consumers, using evaluative and iterative strategies, changing the infrastructure, adapting and tailoring to the context, developing constituent interrelationships, utilizing financial strategies, supporting providers, providing interactive assistance, and training and educating constituents. For example, a preference elicitation of 357 key individuals associated with Philadelphia Medicaid’s behavioral health care system found that constituents generally agreed that financial incentives were the most useful strategy to help clinicians deliver EBTs (Candon et al. 2022, Williams et al. 2021).

Another well-known strategy taxonomy is the Behavior Change Wheel (Michie et al. 2011), which delineates strategies targeting behavior. The Behavior Change Wheel centers around a multilevel behavior system composed of the key conditions of capability, opportunity, and motivation. Around these conditions are nine implementation strategy functions targeting these conditions: education, persuasion, incentivization, coercion, training, restriction, environmental restructuring, modeling, and enablement. These strategies are nestled within seven categories of policy aimed at enabling enactment of the strategies (e.g., communication/marketing, legislation, guidelines, and fiscal measures). The Behavior Change Wheel has been used to guide the selection of implementation strategies for behavioral health EBTs delivered by a range of constituents, including primary care physicians, nurses, community health workers, and contact center staff (Isenor et al. 2021). Both ERIC and the Behavior Change Wheel address the how-to of implementation, but ERIC describes more group- and organization-level strategies while the Behavior Change Wheel is more individual and group oriented. For this reason, the two taxonomies have the potential to be complementary rather than redundant (McHugh et al. 2022).

A key challenge for those who use these taxonomies is selecting the optimal implementation strategies to target contextual barriers and promote successful implementation (Powell et al. 2015). The field of IS has developed a range of tools and methods to facilitate strategy selection. For example, the CFIR-ERIC Implementation Strategy Matching Tool (CFIR Res. Team 2024) maps strategies from the ERIC taxonomy onto identified implementation barriers from CFIR to support the selection of context-appropriate strategies. The CFIR-ERIC matching tool helps narrow 2,847 possible barrier–strategy combinations to a more manageable set identified by IS experts (Waltz et al. 2019). Beyond matching tools, other strategy selection methods include implementation mapping, conjoint analysis, group model building, concept mapping, and partner-driven strategy generation, each of which is described in greater detail elsewhere (Fernandez et al. 2019, 2023; Powell et al. 2017). Across these methods, the strategy selection process must center the specific needs of communities, rather than adopting a one-size-fits-all approach, to ensure the implementation strategies will be feasible, acceptable, and effective in promoting equitable access to the EBT.

To promote generalizable knowledge, implementation strategies must be well specified to support future constituents seeking to apply the findings. According to seminal guidance from Proctor et al. (2013), implementation strategies should be named, defined, and operationalized across seven dimensions: actor, action, action targets, temporality, dose, implementation outcomes addressed, and theoretical justification. Tools have been developed to support the specification and tracking of implementation strategies via Excel spreadsheets (e.g., the Pragmatic Implementation Reporting Tool; Rudd et al. 2020), Redcap data capture platforms (e.g., the Longitudinal Implementation Strategy Tracking System; Smith et al. 2023a), and online software (e.g., the HIV implementation initiative at Northwestern University; see https://hivimpsci.northwestern.edu). The sidebar titled “Step 4: Select Implementation Strategies” demonstrates how researchers selected and operationalized implementation strategies in our firearm safety and CM case examples.

### Step 5: Select Implementation Outcomes

In the final step, implementation outcomes that allow for an evaluation of implementation success must be selected (Figure 1a). Implementation outcomes are “the effects of deliberate and purposive actions to implement new treatments, practices, and services” (Proctor et al. 2011, p. 65) and are the most proximal targets of implementation strategies. The relationship between implementation strategies and outcomes is analogous to that between clinical interventions and outcomes. For example, a clinical psychologist may assess the impact of cognitive behavioral therapy (CBT) to treat depression on clinical outcomes, such as changes in depressive symptoms, daily functioning, and quality of life. In parallel, an implementation researcher may assess the impact of providing financial incentives to clinicians delivering CBT to treat depression on implementation outcomes, such as fidelity of the intervention (Schriger & Beidas 2022).

Although various FTMs describe and define implementation outcomes, two implementation evaluation frameworks are commonly used. Supplemental Table 1 presents the components of each of these frameworks and their areas of overlap and differentiation. The Reach, Effectiveness, Adoption, Implementation, and Maintenance (RE-AIM) framework (Glasgow et al. 1999) identifies five broad outcomes to attend to when considering implementation success: reach, effectiveness, adoption, implementation, and maintenance (see Supplemental Table 1 for definitions). For over two decades, this framework has been used to guide pragmatic efforts toward measurable public health benefits (Glasgow et al. 2019). RE-AIM has become increasingly popular to evaluate EBTs: A 2015 systematic review identified 82 unique behavioral health EBTs that had been assessed using at least one RE-AIM dimension (Harden et al. 2015). A second evaluation model by Proctor et al. (2011) identifies eight key implementation outcomes based on a synthesis of the broader implementation literature: acceptability, adoption, appropriateness, cost, feasibility, fidelity, penetration, and sustainability. These outcomes have been used in an array of behavioral health implementation trials: A scoping review (Proctor et al. 2023) identified over 400 studies that assessed one or more implementation outcomes, of which 22.5% were in behavioral health organizations. Both models have been extended to explicitly center health equity, highlighting the need to consider equitable implementation across client, clinician, organization, and community levels to reduce inequities in health care delivery (Baumann et al. 2023, Shelton et al. 2021b) and ensure that implementation efforts are successful for all, with particular attention paid to marginalized communities.

Measuring implementation outcomes is essential not only to establish the effectiveness of implementation strategies but also to evaluate their impact on downstream service systems and clinical outcomes. Conceptually, implementation outcomes may be thought of as a precondition to achieving the desired clinical effect of an intervention. Extending the prior example, if a trial found that CBT did not reduce depression symptoms, a researcher may presume that CBT was not effective. However, alternate explanations could be possible. It is possible that the methods used to supervise clinicians adopting CBT were not acceptable to the organizational leadership, that the supervision was not delivered with fidelity, or that the intervention was not delivered in an equitable manner and only reached those clients with the most resources available to them. Evaluating a range of implementation outcomes enables researchers to more carefully interpret findings and design appropriate follow-up studies.

A final consideration when selecting outcomes is to ensure they are collected via an appropriate study design. As in intervention research trials, randomization is a mainstay of implementation trials (Brown et al. 2017). Whereas trials evaluating clinical outcomes of EBTs typically randomize at the client level, trials evaluating implementation strategies typically randomize at the clinician or organization levels. For this reason, designs such as cluster randomized trials are common and have been used to evaluate behavioral health EBTs in college counseling centers, primary care, and community-based organizations (Becker et al. 2008, Garner et al. 2012, Wilfley et al. 2020). Recognizing the limitations of two-group parallel randomized trials, Miller et al. (2020) described alternate designs that might be suitable for implementation research, including pre-post designs with a nonequivalent control group, interrupted time series, stepped wedge trials (both randomized and nonrandomized), and adaptive trials.

Across these designs, there are also different typologies of trials that can be used to simultaneously capture both client and implementation, using a hybrid effectiveness-implementation approach. The extent to which the clinical outcomes are viewed as primary or secondary typically depends on the level of evidence for the EBT. If evidence is still developing, measures of clinical outcomes will be of primary importance to establish the intervention’s effectiveness, but there is still utility in measuring secondary implementation outcomes to determine if the EBT is feasible, is acceptable, and can be delivered with fidelity. By contrast, if an EBT has extensive evidence, then measures of implementation will be of primary importance, but it is important to also examine secondary clinical outcomes to ensure that the EBT has the desired effects when implemented under real-world circumstances. Resources exist for those interested in learning more about hybrid trials (Curran et al. 2022).

**Figure 2:**
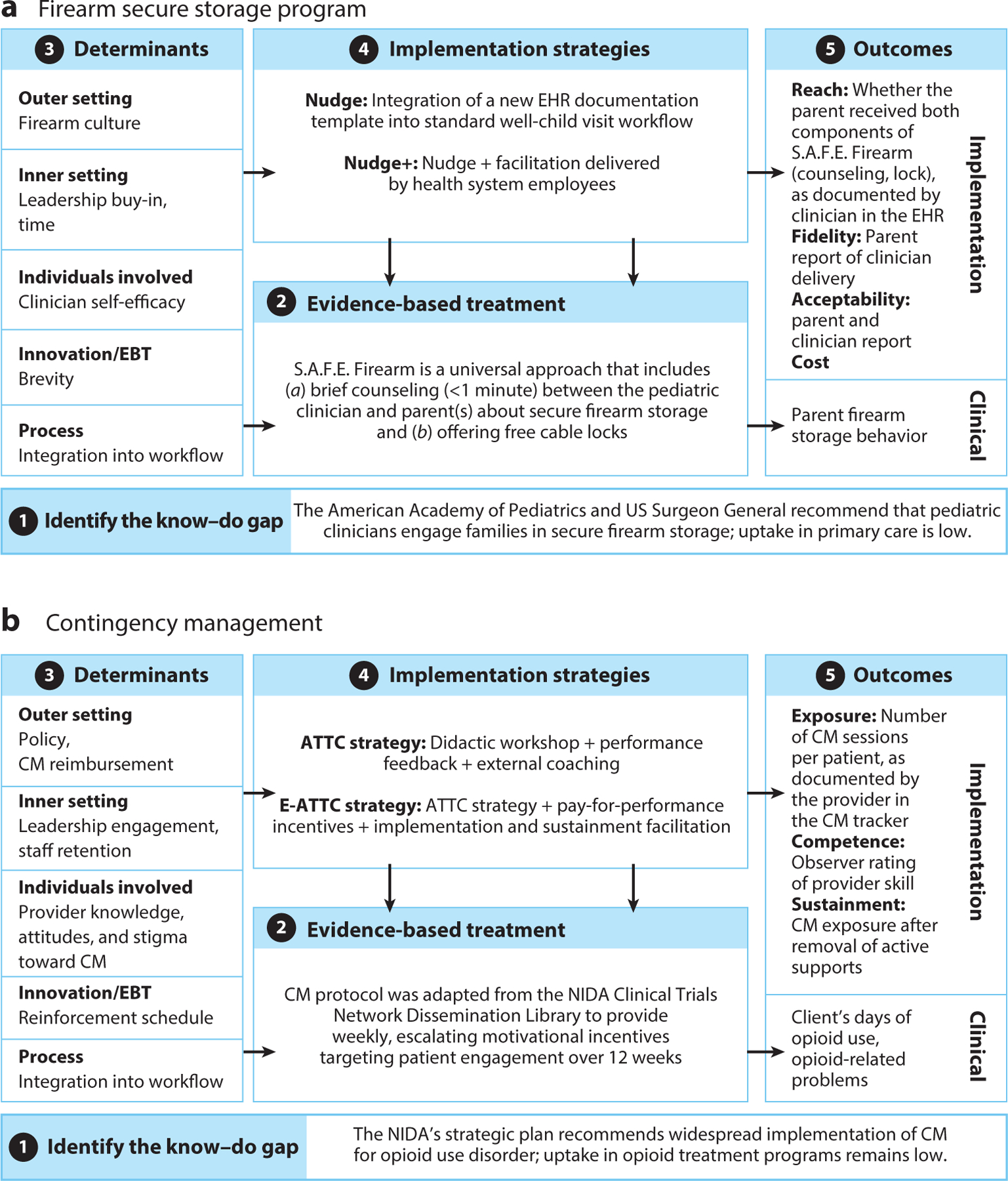
Completed Implementation Research Logic Models for case examples. Circled numbers indicate steps for moving through the figure. Abbreviations: ATTC, Addiction Technology Transfer Center; CM, contingency management; E-ATTC, Enhanced ATTC; EBT, evidence-based treatment; EHR, electronic health record; NIDA, National Institute for Drug Abuse; S.A.F.E., suicide and accident prevention through family education. Figure adapted from Smith et al. (2020) (CC BY 4.0).

The ability to select and specify an appropriate study design for implementation research is an increasingly sought-after area of IS expertise. The sidebar titled “Step 5: Selecting Implementation Outcomes” details the specific outcomes and study designs used in our case examples, and Figure 2 presents all five steps in an IRLM for each case example. The next section highlights related areas of expertise that are needed to build the IS workforce and considers ways in which clinical psychologists are ideally suited to build IS capacity.

## BUILDING CAPACITY IN IMPLEMENTATION SCIENCE FOR CLINICAL PSYCHOLOGISTS

To realize the potential of bridging know–do gaps in behavioral health using the methods described in the previous sections, there is another major gap that must also be addressed: the expertise capacity gap, defined as the gap between the workforce needed and the workforce currently available to accelerate the research-to-practice pathway (Becker et al. 2024). Fortunately, clinical psychologists have foundational competencies that make them particularly well-suited to fill this capacity gap. These core professional competencies include skills in developing and maintaining relationships in interdisciplinary systems, the ability to communicate complex ideas using approachable language, experience selecting and applying EBTs into clinical practice, and scientific research competencies relevant to intervention evaluation designs used earlier in the translational pathway (Proctor & Vu 2019). Such foundational competencies have enabled clinical psychologists and other behavioral health researchers to serve at the forefront of the IS field: They led the call for deployment of EBTs, defined the field of IS (Proctor et al. 2011), and then led the administration of early seminal studies implementing EBTs in real-world settings (Brown et al. 2014).

Several collaborative efforts have articulated core IS competencies needed to bridge the expertise capacity gap. For example, experts and trainees from the National Institutes of Health–funded Mentored Training in Dissemination and Implementation Research in Cancer (MT-DIRC) program developed competencies with a focus on identifying objective indicators of professional development within IS training programs (Padek et al. 2015). Other efforts have included collecting perspectives on competencies from international and multidisciplinary experts (Schultes et al. 2021), implementation practitioners (Tabak et al. 2017), and prospective employers of students graduating from IS courses (Ullrich et al. 2017). Notably, efforts to define IS competencies have applied several of the methods described in earlier sections of this review, such as integrating qualitative and quantitative methods; centering community engagement by including IS practitioners; and applying mapping methods to group competencies by conceptual relationship, importance, and skill level (Metz et al. 2020, Padek et al. 2015, Schultes et al. 2021, Tabak et al. 2017, Ullrich et al. 2017). For example, the MT-DIRC program used a card-sorting method to cluster implementation competencies into developmental skill levels going from basic (i.e., awareness of IS methods) to intermediate (e.g., application of IS methods in research) to advanced (i.e., research focused on advancing IS methods) (Padek et al. 2015).

The number of distinct IS competencies identified by IS researchers has varied (between 7 and 43), as has the number of identified conceptual domains (between 3 and 9), yet a set of core conceptual domains consistently emerges: (*a*) defining, applying, and evaluating IS FTMs and related concepts; (*b*) understanding the context of specific settings or community characteristics; (*c*) selecting appropriate IS study designs (e.g., quasi-experimental, observational); and (*d*) gaining appropriate academic skills, including scientific communication and grants-personship. Several user-friendly manuscripts and tools have been developed to help clinical psychologists and other newcomers to build IS competences in areas such as locating their empirical question on an “IS subway” (Lane-Fall et al. 2019), defining key IS terms, selecting an appropriate study design (Brown et al. 2017, Curran 2020, Hwang et al. 2020), and writing compelling IS grants (Brownson et al. 2015, Crane et al. 2023, Proctor et al. 2012).

Clinical psychologists and implementation researchers share an increasing focus on reducing the expertise capacity gap for conducting equity-oriented research (Baumann et al. 2023, Raque et al. 2021, Shelton et al. 2021b). Yet, despite the centering of equity as a requisite for IS to have its intended impact, a recent survey suggests the field falls short of its perceived capability to meet this need (Baumann et al. 2023). A key factor contributing to this shortcoming is the fact that many training programs do not emphasize the skills and competencies needed to promote IS, and those that do rarely adopt an explicit focus on equity. Infusion of equity-oriented IS competencies into graduate programs and clinical psychology internships can further the capacity of graduates to make a public health impact (Atkins et al. 2014). Relevant equity-oriented IS clinical opportunities include direct care and supervisory, leadership, and management experiences embedded within health systems and community-based treatment settings that emphasize the use of replicable, generalizable methods for all clients (Atkins et al. 2014). Additional opportunities for equity-oriented IS training beyond graduate training include training grants, specialty fellowships, IS training centers, mentorship, conferences, and networking. Such resources have been described in depth in comprehensive reviews for behavioral health researchers (Chambers et al. 2020) and implementation researchers more broadly (Davis & D’Lima 2020, Juckett et al. 2022).

Finally, there is an increasing call for educational initiatives to integrate training focused on implementation science and practice, in recognition of the fact that these are complementary ends of a studying-to-doing continuum with significant overlap and dependency (Leppin et al. 2021). Currently, many educational programs are positioned to train implementation or clinician researchers (e.g., degree-granting graduate programs) or practitioners (e.g., continuous professional development). Few institutions adequately teach both, and even fewer can train researchers and practitioners in a way that prepares them to work together as collaborators within the same clinical and community contexts. Of note, there are a number of leading innovative models that have been developed to support a “hub and spoke” approach to the capacity building of researchers and practitioners, including the Research Adoption Support Center (NIDA grant U2CDA057717), the Ending the HIV Epidemic Implementation Science Coordination Initiative (NIH grant P30AI117943), and the Sociostructural Implementation Science Coordination Initiative (NIH grant R24MH134305), all of which are led or co-led by Northwestern University.

## OPPORTUNITIES AND FUTURE DIRECTIONS IN THE FIELD

In this section, we outline a set of key opportunities for clinical psychologists to continue to lead the field of IS. First, in addition to addressing the research-to-practice and the expertise capacity gaps, clinical psychologists are needed to address a final gap that plagues the field: a methodological innovation gap. In the same manner that clinical psychologists have innovated in intervention science to examine how, when, and for whom interventions work (Kazdin 2007), innovation is needed in IS to understand how, when, and for whom implementation strategies work (Kazdin 2007; Klasnja et al. 2024; Lewis et al. 2018, 2020). Understanding the mechanisms of change underlying implementation strategies can help us to better match our implementation strategies to the contextual determinants and to create integrative causal theories of implementation to advance the field.

Second, in parallel with efforts to enhance methodological innovation, there is an urgent need to enhance the pragmatic, real-world impact of our work. In recent years, there has been recognition of a growing divide between implementation research and practice, with the field potentially replicating the same disconnect between research and practice that it was intended to solve (Beidas et al. 2022). In addition to the need to build IS competencies in the workforce, potential solutions to this divide include the employment of more embedded implementation researchers by health systems and community organizations as well as continued emphasis on the development of pragmatic tools that can be employed by implementation practitioners and behavioral health clinicians. Many of the FTMs and taxonomies introduced in this article are comprehensive yet challenging to apply for individuals new to the field. For instance, the CFIR has 50+ determinants, and the ERIC taxonomy has 73 strategies. For the field to have optimal impact, implementation researchers need to create simple, pragmatic tools that are transferrable across disease states, settings, and patient populations. We believe the IRLM introduced in this article is an example of a relatively simple model, which was developed by three behavioral health researchers. Finally, with their knowledge of human behavior, clinical psychologists are ideally suited to identify novel methods to promote behavior change in providers and organizations. Clinical psychologists, perhaps more than any other scholars, recognize that it is not only the content of an EBT that matters but also how it is delivered (Messer & Wampold 2002). Yet, in IS, the delivery of implementation strategies and the nonspecific factors of implementors that influence change have been relatively unexplored. We believe that clinical psychologists are uniquely well suited to drive methodological innovation, the creation of pragmatic tools, and the identification of novel methods to support behavior change in clinicians working within organizational constraints.

Next, we consider a range of emergent areas that present opportunities for clinical psychologists to further advance the field. These areas represent some of the most exciting future directions in IS.

### Dissemination

Even when EBTs are effectively implemented, they will remain on the shelf if clients are not aware of them and do not access them. Limited research has examined the dissemination of EBTs, or the practices that increase awareness of and demand for innovations effectively and equitably. Dissemination science refers to the scientific study of ways to disseminate or distribute information about innovations, whereas dissemination practice focuses on the activities that spread and/or diffuse knowledge to various audiences (e.g., policymakers, the public). Illustrative examples of dissemination research include work with parents of anxious youth (Crane et al. 2021) and with parents of youth at risk of substance use (Becker 2015; Becker et al. 2018, 2020). Additional research is needed on the determinants of dissemination, strategies to disseminate EBTs, and dissemination outcomes.

### De-Implementation

While the focus on implementing underused EBTs is laudable, equal attention must be placed on the concept of de-implementation, or supporting change efforts focused on decreasing use of non-EBTs (Walsh-Bailey et al. 2021). It is important to study both underuse of EBTs and overuse of non-EBTs, particularly considering the need to ensure that workflows are optimized, clinician burden is minimized, and low-value and iatrogenic treatments are replaced. For example, historically, clinical psychology has promoted iatrogenic treatments for people from sexual and gender minority communities (Comer et al. 2024). Now that we understand that these treatments are harmful, unethical, and not aligned with modern standards for clinical practice, de-implementation of such practices is critical.

### Sustainment

As outlined in the EPIS framework (Aarons et al. 2011), implementation is phasic, and after a clinician or organization implements an EBT for some time, the end goal is the routinization or maintenance within that context—that is, transitioning from a change effort to business as usual (Shelton et al. 2018). Currently, little implementation research focuses on this phase (Moullin et al. 2019). It is likely that active approaches to sustain EBTs are needed (Nathan et al. 2022), and new literature suggests that there are three broad approaches: self-sustainment (i.e., implementation of the EBT is expected to continue without additional support), sustainment support (i.e., implementation of the EBT continues with support), and dynamic sustainment support (i.e., implementation of the EBT continues with dynamic support that evolves over time via intentional adaptation) (Wolfenden et al. 2024). To truly understand ongoing implementation and sustainment, evaluation of implementation must continue over long periods of time. Illustrative behavioral health examples include work in Philadelphia (Last et al. 2023) and Los Angeles County (Brookman-Frazee et al. 2016).

### Implementation of Digital Mental Health Interventions

Given the global behavioral health crisis and the need to scale up interventions to reach all those who are in need (Rudd & Beidas 2020), digital mental health has been touted as a potential solution. While offering an exciting new direction, much of the challenge related to achieving this vision relates to client engagement with these technologies (Borghouts et al. 2021) as well as implementation challenges (Smith et al. 2023b). Clinical psychologists have led work examining some of the unique IS considerations plaguing the scalability of digital behavioral health tools (Graham et al. 2020, Hermes et al. 2019).

### Policy Implementation

Policy IS is an emerging subarea (Emmons et al. 2021). This work can include policy focusing on (*a*) the EBT to adopt and implement, as in the example of policy implementation in US states to improve substance use disorder treatment (Crable et al. 2022); (2) the context in which implementation operates, as in the example of Lengnick-Hall et al.’s (2021) study linking outer and inner contexts during implementation and sustainment; and (*c*) the strategy to support an EBT (Purtle et al. 2023), as explored in Beidas et al.’s (2019b) naturalistic observational study in the city of Philadelphia.

### Unintended Consequences of Implementation

The core of IS is to improve care by increasing the reach of EBTs. However, IS can also result in unintended consequences that are often overlooked (Beidas et al. 2022). There is an opportunity for more thoughtful and prospective planning for, and evaluation of, unintended consequences within implementation research. Pullmann et al. (2022) conducted a survey of key constituents in pediatric and adolescent mental health, offering 13 categories of unintended consequences that may serve as a roadmap to prospectively plan, select, and adapt implementation strategies.

### Embedding Equity and Social Justice into All Efforts

Following the racial reckoning in the United States in 2020, the discipline of IS has called for application of insights from the established discipline of health equity (Baumann & Cabassa 2020, Brownson et al. 2021, Shelton et al. 2021b). A first step in this approach is to ensure that implementation efforts do not result in inequitable reach, thus reifying existing inequities or creating new inequities. For example, Hoskins et al. (2022) embedded an equity evaluation into a pilot trial preceding the comparative effectiveness implementation trial in the example of the secure firearm storage program described throughout this article. Recent work has called for a move beyond evaluating equity to employing IS as a tool to advance social justice (Bradley et al. 2023; Shelton et al. 2021a,b) and ensuring that all IS work is conducted with an actively antiracist lens. We argue that there is no implementation without centering equity and antiracism.

### Implementation of Structural Interventions

Related to the need to advance equity, it is clear that many health disparities observed in the United States are a consequence of structural determinants such as racism (Acker et al. 2023, Mohebbi et al. 2024), discrimination (Lei et al. 2021), and poverty (Ridley et al. 2020). Thus, further study of the implementation of interventions that address these structural determinants is an important area of future inquiry (Dodge et al. 2024, McGinty et al. 2024) in which IS must play a mission-critical role (McGinty et al. 2024). Such work can include large-scale hybrid effectiveness-implementation studies of structural interventions (e.g., place-based interventions such as greening; South et al. 2018).

### Coming Together as a Science

The past two decades have seen a proliferation of work focused on closing the know–do gap. Much of that work has focused on legitimization of the science and development of frameworks, taxonomies, and outcomes. IS integrates best practices from other fields including management, economics, and engineering. Given the increasing number of individuals working in this area, it is time for us all to come together with our colleagues working in adjacent and aligned areas including improvement science, health care delivery science, and health equity (Chambers & Emmons 2024). This includes a need for work occurring globally under this umbrella, which often occurs in a siloed manner, to align and synergize (Beidas et al. 2022). This will allow us to develop a more powerful voice and strengthen the potential impact of our learnings. For example, IS was not represented at the table with the US COVID-19 taskforce, and it is not always considered in relation to the move to deploy globally digital mental solutions to meet need at scale. Given the maturation of our science, there is a need to align and synergize with adjacent fields.

## CONCLUSION

Clinical psychologists can and do play a leadership role in closing the know–do gap in behavioral health and beyond. The unique skill set that psychologists possess as experts in human behavior change allows us to apply the same steps used in psychotherapy to change efforts at the clinician, organization, and system levels. The next frontier of implementation research will advance methods to increase use of EBTs, decrease use of non-EBTs, and ensure that all people benefit from scientific discoveries in behavioral health.

## Supplementary Material

SM

## Figures and Tables

**Table 1:** Overview of the Exploration, Preparation, Implementation, and Sustainment (EPIS) framework and alignment with phases of treatment delivered by clinical psychologists

EPIS phase	Definition	EPIS phase goals and milestones	Alignment with treatment phases for clinical psychologists
Exploration	Exploration refers to the process of working closely with community constituents to evaluate and identify clinician, organizational, and community needs and select potential EBTs to meet their needs and fit the setting where implementation will ultimately occur.	∎ Conduct ongoing meetings, consensus building discussions, and outreach ∎ Strengthen partnerships between researchers and community constituents ∎ Select programs to be implemented that align with community constituents’ needs	∎ Conduct initial session with client to build rapport ∎ Select EBT to be used with the client that aligns with the client’s needs based on a comprehensive case conceptualization
Preparation	Preparation involves the crucial prework of conducting a needs assessment focused on identifying needed EBT adaptations, assessing contextual determinants impacting EBT implementation, and selecting implementation strategies.	∎ Continue ongoing meetings and discussions between researchers and community constituents ∎ Conduct iterative mixed-method (i.e., qualitative and quantitative) needs assessment with constituents to assess EBT adaptations and potential implementation barriers and facilitators ∎ Select implementation strategies to support scaling up of EBTs in collaboration with constituents ∎ Deploy selected implementation strategies as needed prior to implementation (e.g., didactic training, social influence and motivation building techniques).	∎ Schedule treatment sessions with a regular, agreed-upon frequency and duration ∎ Conduct iterative biopsychosocial assessment to evaluate client needs, presenting problems/diagnoses, EBT adaptations, and barriers/facilitators to client engagement in treatment ∎ Select specific EBT practice elements to address client’s presenting problems/treatment targets, in collaboration with client ∎ Deploy selected EBT practice elements as needed prior to delivering EBT (e.g., treatment rationale, psychoeducation)
Implementation	Implementation focuses on taking steps to implement and scale the EBT through the deployment and monitoring of the implementation strategies selected during the preparation phase.	∎ Continue ongoing meetings and discussions between researchers and community constituents ∎ Deploy implementation strategies selected in the preparation phase ∎ Track delivery of implementation strategies ∎ Regularly collect clinical effectiveness (e.g., client symptom change) and implementation outcome (e.g., EBT fidelity) data at the client, clinician, and clinic levels	∎ Continue scheduled treatment sessions with a regular, agreed-upon frequency and duration ∎ Deploy the planned EBT practice elements with client ∎ Track EBT practice elements delivered ∎ Regularly collect clinical effectiveness (e.g., client symptom change) data at the client level
Sustainment	The sustainment phase aims to support community constituents to continue EBT implementation with fidelity, often without ongoing support from the research team or other support teams (e.g., EBT expert purveyors).	∎ Taper and end ongoing meetings and discussions between researchers and community constituents ∎ Warm handoff of EBT implementation to community constituents ∎ Continue to track delivery of implementation strategies ∎ Continue to collect clinical effectiveness and implementation outcome data at the client, clinician, and clinic levels	∎ Taper and end treatment sessions and prepare for client discharge ∎ Warm handoff of skills learned in session to the client for ongoing practice and use ∎ Terminate treatment

Abbreviation: EBT, evidence-based treatment.

## STEP 1: IDENTIFY THE KNOW–DO GAP

### Practice of Interest #1: Firearm Secure Storage Program

The know–do gap was between the recommendations of leading organizations such as the American Academy of Pediatrics that pediatric clinicians engage families around secure firearm storage as part of safety discussions (e.g., pool safety, helmets, seatbelts) and the low uptake of these recommendations in practice.

### Practice of Interest #2: Contingency Management

The know–do gap was between the evidentiary support for CM, an EBT that provides clients with incentives for meeting treatment goals, and its extremely low uptake in opioid treatment programs. This gap was so pronounced that the NIDA specifically referenced the evidence for CM and the need to advance its widespread implementation in its strategic plan (NIDA 2022).

## STEP 2: SELECT AN EVIDENCE-BASED PRACTICE

### Practice of Interest #1: Firearm Secure Storage Program

An existing EBT included screening for firearms, counseling families about securing firearms, and providing cable locks (Barkin et al. 2008). The implementation science team (Wolk et al. 2018) engaged constituents in adaptation (Davis et al. 2021). The constituents shared concerns that firearm ownership might be recorded in the electronic health record and raised the importance of universal discussions about secure firearm storage, given high rates of firearm ownership. Thus, screening was removed, and all families were counseled on firearm storage and offered free locks. Finally, firearm- and non-firearm-owning families were queried about the acceptability of the adapted program (Hoskins et al. 2021).

### Practice of Interest #2: Contingency Management

NIDA-funded researchers developed a CM protocol, including guidance on duration, frequency, and reinforcement. The implementation science team (Becker et al. 2019b) engaged constituents from 11 opioid treatment programs to adapt NIDA’s CM protocol. This collaborative process solicited feedback on the preferred behavioral targets, the staff to administer CM, and the duration and frequency of reinforcement. Feedback revealed preferences for CM to be administered within individual counseling sessions by the primary counselor at the opioid treatment program. A 12-week CM protocol with weekly, escalating motivational incentives targeting patient engagement was specified (Becker et al. 2021).

## STEP 3: IDENTIFY CONTEXTUAL DETERMINANTS

### Practice of Interest #1: Firearm Secure Storage Program

The team mapped out all constituent groups (Wolk et al. 2017): parents, physicians, nurses and nurse practitioners, leaders of pediatric primary care practices, behavioral health and quality improvement experts, system leaders, third-party payers, members of national credentialing bodies, and firearm constituents. The team took a mixed-methods approach, first surveying pediatric clinicians to determine the (*a*) acceptability and (*b*) utility of intervention components (Beidas et al. 2019a). The team then interviewed constituents to understand their perspectives on the CFIR determinants: innovation characteristics (e.g., brevity), inner setting (e.g., leadership buy-in, time), outer setting (e.g., firearm culture), individuals involved (e.g., clinician self-efficacy), and implementation processes (e.g., need to integrate within existing workflows) (Wolk et al. 2018, Jager-Hyman et al. 2019).

### Practice of Interest #2: Contingency Management

After specifying the CM protocol, the team reviewed the literature to identify determinants. There has been extensive commentary on barriers to and facilitators of CM implementation in both the peer-reviewed literature (Becker et al. 2019a, Rash et al. 2020) and the popular press (Goodnough 2020). Additionally, the team used in-depth interviews to solicit barriers to and facilitators of CM implementation (Becker et al. 2019b). Results identified an array of determinants corresponding with the CFIR domains: innovation characteristics (e.g., complexity of reinforcement), inner setting (e.g., leadership engagement, staff retention/turnover), outer setting (e.g., policy, CM, reimbursement), individuals involved (e.g., provider knowledge, attitudes, and stigma toward CM), and implementation processes (e.g., need to integrate CM into existing workflows).

## STEP 4: SELECT IMPLEMENTATION STRATEGIES

### Practice of Interest #1: Firearm Secure Storage Program

Based on the inputs from the previous steps, we applied implementation mapping (Beidas et al. 2021). The team identified the following implementation strategies: (*a*) an EHR-based strategy and (*b*) facilitation to address the constituents’ suggestions regarding brevity, firearm culture, workflow integration, leadership, and self-efficacy. The first strategy prompted clinicians to deliver the intervention (i.e., nudge) by modifying the choice architecture of the EHR (Thaler & Sunstein 2008). The second strategy, facilitation, or problem-solving support to each clinic, was delivered by health system employees external to the clinics for less than 1 hour a month over 1 year.

### Practice of Interest #2: Contingency Management

Researchers selected strategies based on the empirical evidence. Prior work showed that a multilevel strategy used by Addiction Technology Transfer Centers (ATTCs) (didactic training plus performance feedback plus external coaching) was significantly more effective in increasing CM adoption than didactic training only (Becker et al. 2016). However, results indicated opportunities to improve acceleration and sustainment of CM adoption (Becker et al. 2016, Helseth et al. 2018). The research team devised an Enhanced ATTC strategy, integrating two theory-driven implementation strategies: (*a*) pay-for-performance incentives, theorized to accelerate CM uptake by targeting implementation climate; and (*b*) implementation and sustainment facilitation, theorized to sustain CM uptake by targeting leadership engagement (Becker et al. 2021). Facilitation was delivered to organizational leaders via monthly hour-long sessions for 9 months (Hartzler et al. 2023).

## STEP 5: SELECTING IMPLEMENTATION OUTCOMES

### Practice of Interest #1: Firearm Secure Storage Program

Because the secure firearm storage program was tested previously in an effectiveness trial, a hybrid type III trial was selected. The trial prioritized measurement of implementation outcomes and collected clinical outcomes as secondary outcomes (Beidas et al. 2021). The implementation outcomes of interest were reach (i.e., clinician report of how many parents had received the intervention), fidelity (i.e., parent report of clinician delivery of the intervention), cost, and acceptability (based on parent and clinician report) (Beidas et al. 2021). Clinical outcomes included parent firearm storage behavior (see Figure 2a for the complete IRLM).

### Practice of Interest #2: Contingency Management

Similar to the first case example, this project used a hybrid type III design that prioritized measurement of implementation outcomes and collected clinical outcomes as secondary indicators, due to the wealth of evidence documenting the effectiveness of CM (Becker et al. 2021). Outcomes were assessed via a 2-group, 28-site cluster randomized trial that tested a standard ATTC condition versus an Enhanced ATTC condition that integrated two theory-driven strategies. Implementation outcomes included CM exposure (i.e., provider report of number of CM sessions per patient), CM competence (i.e., observer rating of provider skill of CM delivery using recorded sessions), and CM sustainment (sustained CM delivery after removal of active support) (Becker et al. 2021). Clinical outcomes included patient days of opioid use and patient opioid-related problems (see Figure 2b for the complete IRLM).

## SUMMARY POINTS

1. Implementation science is the discipline focused on closing know–do gaps and producing generalizable knowledge about effective methods to equitably increase the availability, adoption, and sustainment of evidence-based psychological treatments, with the end goal of improving population health.
2. We present a blueprint for applying implementation science approaches to clinical psychology research and practice, using two illustrative examples to bring the process to life: secure firearm storage and contingency management.
3. To lead in the application of implementation science, capacity building is needed, and clinical psychologists have foundational competencies that are particularly well-aligned with this kind of work.
4. There are key opportunities for further innovation that can be led by clinical psychologists, including the dissemination and sustainment of evidence-based treatments, the de-implementation of ineffective or harmful practices, and the centering of health equity in implementation research, practice, and capacity-building efforts.

## Footnotes

1 Readers are referred to the “Research Priorities: Dissemination & Implementation (D&I) Research” page of the National Institutes of Health’s website at https://prevention.nih.gov/research-priorities/dissemination-implementation.

## Abbreviations

NIMH: National Institute of Mental Health
EBT: evidence-based treatment
NIDA: National Institute for Drug Abuse
IS: implementation science
FTMs: frameworks, theories, and models
EPIS: Exploration, Preparation, Implementation, Sustainment
IRLM: Implementation Research Logic Model
CM: contingency management
CFIR: Consolidated Framework for Implementation Research
HEIF: Health Equity Implementation Framework
ERIC: Expert Recommendations for Implementing Change
CBT: cognitive behavioral therapy
RE-AIM: Reach, Effectiveness, Adoption, Implementation, and Maintenance
MT-DIRC: Mentored Training in Dissemination and Implementation Research in Cancer
