# Supplementary material for: Harnessing Implementation Science in Clinical Psychology: Past, Present, and Future
Source: Annu Rev Clin Psychol. Author manuscript; Available in PMC 2025 Jul 11. (PMC12247127; doi:10.1146/annurev-clinpsy-081423-021727)
Supplement: SM [file NIHMS2089168-supplement-SM.pdf]

Supplemental Table 1. Points of Overlap and Differentiation in Two Seminal Implementation Evaluation Frameworks.

Outcome Type	Outcome	Proctor	RE-AIM	Comparison and Synthesis	Evaluation of Outcomes to Center Equity
**Perceptual**	*Acceptability*	Perception that an EBT is agreeable or satisfactory (e.g., "I like it!")	Analogous construct not included in RE-AIM	Although acceptability, appropriateness, and feasibility are not included in RE-AIM or CFIR, some researchers assert that perceptions of acceptability, appropriateness, and feasibility are key preconditions for the initial decision to adopt an EBT. Once a setting and provider workforce have experience with an EBT, perceptions of acceptability, appropriateness, and feasibility are key preconditions for sustainment/maintenance	Do perceptions of acceptability, feasibility, and appropriateness differ by community, organizational, clinician, and client characteristics?
**Perceptual**	*Appropriateness*	Perception of fit, relevance, or compatibility of an EBT for a setting, therapist, or patient; perception of the fit of the EBT to address a specific clinical problem (e.g., "It's a good fit!")	Analogous construct not included in RE-AIM		
**Perceptual**	*Feasibility*	Perception of the extent to which the EBT can be successfully used in a given setting (e.g., "We can do it!")	Analogous construct not included in RE-AIM		
**Behavioral**	*Adoption*	The intention, initial decision, or action to try or employ an innovation or evidence-based practice.	The absolute number, proportion, and representativeness of individuals who are willing to make the initial decision to participate in a given initiative, intervention, or program	The Proctor framework frames the definition of adoption in terms of clinician level whereas RE-AIM frames it in terms of both the clinician and organizational/setting level.	Does adoption differ by community, organization, clinician, and client characteristics? Is adoption equitable across communities, organizations, clinicians, and clients who could stand to

Downloaded from www.annualreviews.org. Guest (guest) IP: 75.210.201.142 On: Fri, 20 Jun 2025 13:16:39

					benefit from the EBT?
**Behavioral**	*Fidelity/ Implementation*	**Fidelity:** the degree to which an EBT is implemented as it was intended	**Implementation:** at setting level, fidelity to the components of an EBT protocol, the time and cost of the EBT; at the patient level, patients' use of the EBT strategies and skills	The Proctor framework tends to frame implementation/fidelity in terms of the clinician-level whereas, RE-AIM includes implementation/fidelity at the organizational and patient levels; RE-AIM also folds cost and time considerations into this construct whereas the Proctor Framework treats it as its own construct.	Does fidelity to the EBT differ by community, organizational, clinician, and client characteristics?<br><br>Is fidelity of care equitable across communities, organizations, clinicians, and clients who could stand to benefit from the EBT?
**Behavioral**	*Sustainment/ Maintenance*	**Sustainment:** extent to which an EBT is institutionalized within a setting	**Maintenance:** extent to which an EBT is institutionalized within a setting; at the patient level, long-term effects of the EBT after 6 or more months after the most recent intervention contact.	At the setting level, Proctor and RE-AIM provide nearly identical definitions of sustainment/maintenance. RE-AIM adds a definition of maintenance at the patient level. CFIR acknowledges both organizational and provider decisions to sustain. CFIR adds the distinction of the anticipated outcome (sustainability) versus actual outcome (sustainment).	Does sustainment of the intervention differ by community, organizational, clinician, and client characteristics?<br>Is sustainment equitable across communities, organizations, clinicians, and clients who could stand to benefit from the EBT?
**Impact**	*Penetration/ Reach*	**Penetration:** integration of a practice within a service setting and its subsystems	**Reach:** number, proportion, and representativeness of individuals who are willing to participate in	Both Penetration and Reach capture those involved in the EBT (clinician); Reach also emphasizes a comparison of the characteristics of	Does reach differ by community, organizational, clinician, and client characteristics? Is

Downloaded from www.annualreviews.org. Guest (guest) IP: 75.210.201.142 On: Fri, 20 Jun 2025 13:16:39

Downloaded from www.annualreviews.org. Guest (guest) IP: 75.210.201.142 On: Fri, 20 Jun 2025 13:16:39

			(clinicians) or receive (clients) an EBT	those who do and do not receive the EBT (clients)	reach equitable across communities, organizations, clinicians, and clients who could stand to benefit from the EBT?
**Impact**	*Effectiveness*	Construct categorized as a service outcome in Proctor's framework	The impact of an EBT on client outcomes (e.g., symptom change, quality of life, negative effects)	Not applicable. The Proctor framework focuses solely on implementation and does not include effectiveness, whereas RE-AIM includes both.	Are some groups of clients experiencing greater benefit from the EBT than others? Are some groups of clients experiencing negative effects from the EBT?
**Impact**	*Cost*	Cost of implementing and delivering the EBT	Subsumed in the implementation construct in RE-AIM	The Proctor framework explicitly calls out cost, specifying that overall costs include the cost of the EBT, the strategies used, and the location of delivery. RE-AIM traditionally includes cost under implementation, although increasingly researchers have applied measurement of cost across each of the five framework dimensions.	Does the cost of implementing differ based on community, organizational, clinician, and client characteristics? Does the cost represent a greater financial burden for some sites compared with others?

Note. Perceptual = based on an individual's perspective/experience, Behavioral = objectively quantifiable, Impact = resultant outcome from implementation at organization and individual levels (Lyon & Bruns 2019). Comparisons of RE-AIM (Glasgow et al 2019) and the framework by Proctor et al (2011) were informed by Reilly et al (2020), as well as commentary in the CFIR Outcomes Addendum (Damschroder et al 2022).

REFERENCES

Damschroder LJ, Reardon CM, Widerquist MAO, Lowery J. 2022. Conceptualizing outcomes for use with the Consolidated Framework for Implementation Research (CFIR): the CFIR Outcomes Addendum. *Implement. Sci.* 17:7

Glasgow RE, Harden SM, Gaglio B, Rabin B, Smith ML, et al. 2019. RE-AIM planning and evaluation framework: adapting to new science and practice with a 20-year review. *Front. Public Health* 7:64

Lyon AR, Bruns EJ. 2019. User-centered redesign of evidence-based psychosocial interventions to enhance implementation—hospitable soil or better seeds? *JAMA Psychiatry* 76:3–4

Proctor E, Silmere H, Raghavan R, Hovmand P, Aarons G, et al. 2011. Outcomes for implementation research: conceptual distinctions, measurement challenges, and research agenda. *Adm. Policy Ment. Health* 38:65–76

Reilly KL, Kennedy S, Porter G, Estabrooks P. 2020. Comparing, contrasting, and integrating dissemination and implementation outcomes included in the RE-AIM and implementation outcomes frameworks. *Front. Public Health* 8:430

Downloaded from www.annualreviews.org. Guest (guest) IP: 75.210.201.142 On: Fri, 20 Jun 2025 13:16:39
